# Treating peri-trochanteric hip fractures with intramedullary nail, when a second anti-rotational screw is necessary?

**DOI:** 10.1186/s12891-023-06892-y

**Published:** 2023-10-04

**Authors:** Panagiotis Tilaveridis, Efthymios Iliopoulos, Paraskevas Georgoulas, Georgios Drosos, Athanasios Ververidis, Konstantinos Tilkeridis

**Affiliations:** 1 Trauma & Orthopaedics Department, General Hospital of Dramas, Drama, Greece; 2 Trauma & Orthopaedics Department, University Hospital of Alexandroupolis, Democritus University of Thrace, Alexandroupolis, Greece

**Keywords:** Hip fractures, Unstable intertrochanteric fractures, Antirotation hip screw, Fracture complications, Fragility fractures

## Abstract

**Introduction:**

Fragility fractures around the proximal end of the femur have increased in recent years due to an aging population, adding to the pressure on national health care systems and to hospital expenses. Peri-trochanteric fractures have historically been treated successfully with anatomic intramedullary nails, giving stable fixation in order to allow early for mobilisation of these frail patients. Some of these nails allow a second (anti-rotational) screw through the nail into the femoral head. We assessed the use of this additional screw in terms of quality of reduction, post-operative mobilization and complications.

**Materials & methods:**

All patients who were treated in the same hospital for peri-trochanteric fracture between January 2017 and December 2019 were included in the study. The patients were randomly assigned into group A, where the treatment included intramedullary nailing using one femoral hip screw, and group B, where the treatment additionally included a second anti-rotational screw. The patients were followed up clinically and radiologically, for at least 3 months post-operatively. Demographic and operative data were collected alongside radiographic and clinical data.

**Results:**

A total of 118 patients with an average age of 82.7 years were included in the study after exclusion criteria was applied. There were no statistically significant differences between the two groups regarding the ASA grade, Nottingham Hip fracture score, Koval score, Mental score, operation time, transfusion requirements, and operative radiation dose and time (*p* > 0.05). In group A, more complications were observed (*p* < 0.05). The radiographic measurements were statistically significantly different. CART analysis revealed that the use of a single screw in the femoral head for the subgroup of the unstable peri-trochanteric fractures (Jensen Type 4–5 – AO31A2.2 and above), has a tendency toward developing more post-operative complications, though this was not statistically significant.

**Conclusion:**

The use of an additional anti-rotational screw for unstable peri-trochanteric fractures (Jensen Type 4–5 and AO 31A2.2 and above) could prevent complications such as varus collapse and cut-out.

## Introduction

Hip fractures with their increasing frequency and severity have become a significant burden to the public health care systems worldwide. According to global epidemiological projections, the annual incidence of hip fractures will increase from 1.66 million on 1990 to 6.26 million by 2050 [1]. Almost half of the patients with hip fractures have fractured the peri-trochanteric area of the proximal femur. The treatment of these type of fractures represent a significant economic burden on the US health care system, accounting approximately 2.6 billion US dollars annually [2]. Therefore it becomes clear that even small modifications and improvements in treatment methods and outcomes would benefit a huge number of patients and at the same time bring significant economic benefits to health care systems. Patients with peri-trochanteric fractures, except from preventing the fracture and enhancing their post-operative rehabilitation, can benefit from better quality of reduction and better stabilization methods [3, 4].

The most common surgical treatment options for peri-trochanteric fractures are the internal fixation using the sliding hip screw system with plate and screws (SHS) and the intramedullary nail using a proximal femur intramedullary nail (IM nail). The use of the IM nails have been increasing the last two decades. The main benefits of IM nails are the less traumatic minimally invasive insertion, the shorter operative time and the reduced intra-operative blood loss. Biomechanically the IM nails have reduced lever arm and can substitute the lateral wall of the proximal part of the femur, presenting a theoretical mechanical advantage comparing to the SHS. On the other hand the application of a sliding hip screw system provides a continuous fracture compression while the patient bears weight. In many countries SHS systems have reduced cost and present comparable clinical outcomes with the IM nails [5].

Despite the latest advances of the IM nailing systems, some technical aspects remain uncertain and require more investigation. In some nails, there is an option of applying a second (anti-rotation) lag screw into the femoral head. Biomechanically, the second lag screw provides more stability to the fixation, especially in unstable fracture patterns [6]. It may prevent some complications such as fracture collapse and lag screw sliding [7]. The application of this second screw is not yet studied adequately in the literature, and its use remains in the surgeons’ preference or ‘feeling’ of fracture stability. In this study, we aimed to assesses the use of a second (anti-rotation) lag screw in the femoral head is studied in terms of quality of reduction, mobilization and complications [7, 8].

## Materials and methods

All patients with a peri-trochanteric fracture treated from January 2017 until December 2019, in the same level 2 trauma centre were prospectively included in the study. Patients with fracture patterns feasible to treatment with a short proximal femoral nail were included in the study and randomly assigned into two study groups. The randomisation was conducted by using a computer generated algorithm at the time of admission. Patients in Group A were treated operatively using a short proximal femoral nail (KFN, Königsee Implantate GmbH, Germany) with one lag screw, and patients in Group B were treated using the same nail and the addition of the second anti-rotational lag screw. Patients with subtrochanteric fractures, requiring long nailing or pathological fractures were excluded from the study. The patients who had less than three months follow-up due to death or other reasons were also excluded from the study. The patients were followed up until radiological and clinical union, for at least three months.

**Fig. 1:**
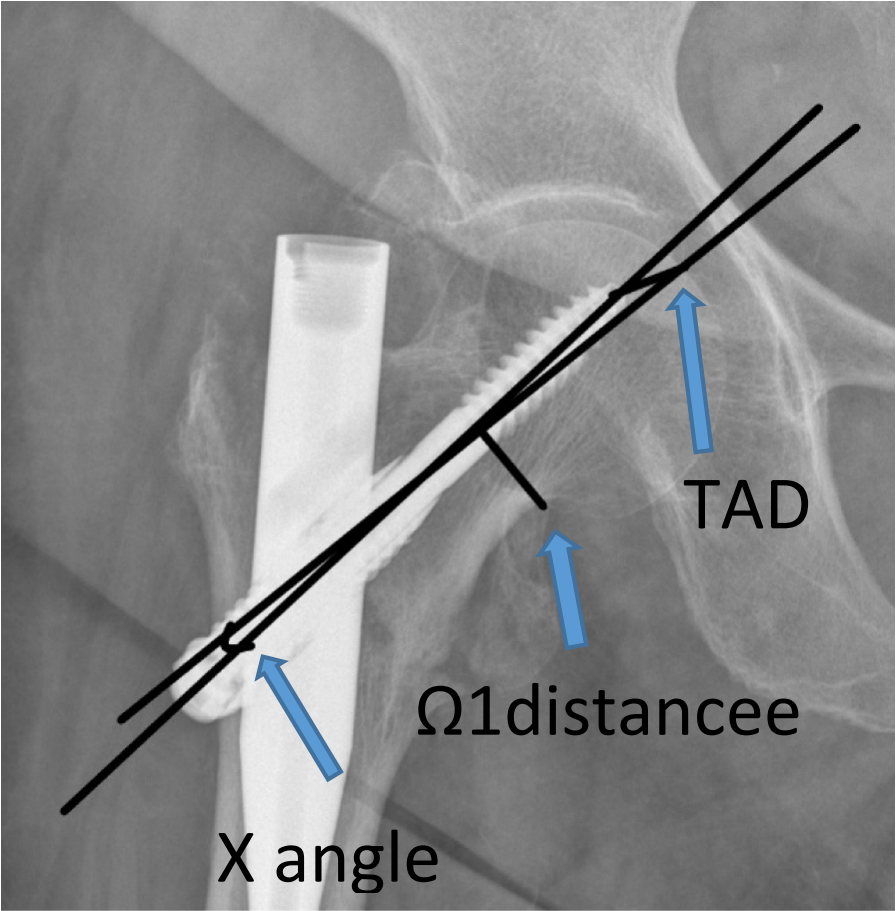
Radiographic measurements obtained from the follow-up radiographs. The diameter of the neck was measured at the point where the roundness of the head ends of the head and the distance of the lag screw from the lower border of the neck and the distance of the anti-rotational nail from the upper border of the neck, for these distances the letter Ω due to its shape, Ω: the diameter of the neck, Ω1: the distance of the lag screw from the lower border of the neck and Ω2: the distance of the anti-rotation nail from the upper border of the neck. The posterior slippage of the lag screw in the first postoperative months was measured as the distance of the lag screw from the lower border of the neck and the TAD give us the position of the nail, i.e. how low and how deep it is located, and the angle X informs about its direction

Preoperative demographic, injury and medical history data were collected. The mental status of the patients was evaluated with the abbreviated mental score [9]. The general medical condition was evaluated and documented using the ASA grade. The Nottingham Hip Fracture score was measured [10], and the general mobility of the patients was assessed using the Koval score [11]. Blood test results pre- and post- operative were documented. Operative data about surgery time and radiation exposure were also collected. All operations were performed by three fellowship trained orthopaedic consultants, in a standardised manner. Under spinal or general anaesthesia, a closed reduction was performed using the traction table. Once an adequate reduction was achieved the application of the intramedullary nail was performed. All patients followed the same post-operative protocol with early mobilisation, weight bearing as tolerated, the day after the operation. The patients were discharged from the hospital once their mobility and their clinical condition was deemed to be adequate. The patients were followed up in the outpatients’ department of the same hospital and radiographic examination was performed during each visit. The pre-operative radiographs were examined by two independent blinded examiners and the fractures were classified using the Jensen and the AO classification [12, 13]. The examiners were then evaluated the post-operative radiographs measured the following parameters: 1) the neck-shaft angle, 2) the reduction gap on the lateral radiograph, 3) the angle formed by the nail with the straight which is parallel to the axis of the neck (X angle) 4) the TAD distance was measured for both lag screws in the antero-posterior and lateral radiographs, 5) the distance of the lag screw from the lower border of the neck, 6) the posterior slippage of the lag screw [14]. (Fig. 1) Complication data such as cut-out, varus mal-reduction, metalwork failure, Z-effect phenomenon, ectopic ossifications were also collected.

### Ethics approval and consent to participate

The study was approved by the local ethics committee of General Hospital of Dramas (Ref. 2502/10–03-2016), and informed consent has been obtained by all patients prior to the participation in the study.

### Statistical analysis

The SPSS version 23.0 statistical programme (SPSS Inc, Chicago, IL, USA) was used for the statistical analysis and the statistical significance was set to < 0.05. In order to identify the most important factors affecting the occurrence of post-operative complications Classification and Regression Trees (CART) were used [15, 16]. CART is a powerful method which can deal effectively with the complexity often inherent in such data sets. They repeatedly divide the data into two mutually exclusive groups on the basis of a single explanatory variable until a set of homogenous groups, in terms of the response variables, is achieved, or until the data cannot be divided any further based on the explanatory variables available. At each division the variable that best divides the data is used. Hence, instead of estimating the mean value of a range of factors associated with the response variable, classification trees identifying specific thresholds of variables above or below which a particular value of the response variable is observed. For this analysis the observation of post-operative complications was used in a nominal scale (0 = no complications, 1 = complications) as the response variable. The explanatory variables employed were the classification of the fracture severity in the Jensen scale, the age of the patient, the gender, ASA, Nottingham, the mental condition of the patient and of course the presence or not of the second anti-rotational lag screw (group A and B).

Among more than 20 Classification Trees, the most suitable was selected based on two criteria. First, the selected tree should have the lowest misclassification rate and highest kappa statistic which should exceed 0.4 [17, 18]. An “honest” misclassification rate was achieved by using tenfold cross validation. Accordingly, the dataset of 118 samples was split into 10 approximately equal partitions, each of which in turns was used for testing while the remaining was used for training the classifier. The validation procedure was repeated ten times and consequently each sample was used nine times for training the classifier and once for testing it [16]. The second criterion was based on the comparison between the correct classification rate of the tree and the respective rate of the “null model” which in this case was 80.5%. The results obtained using CART were further confirmed using a Chi-Square analysis for all cases where the Jensen score of fracture severity was above or equal to 4 (Unstable fractures according to the AO classification – AO 31A2.2 and higher).

## Results

**Fig. 2:**
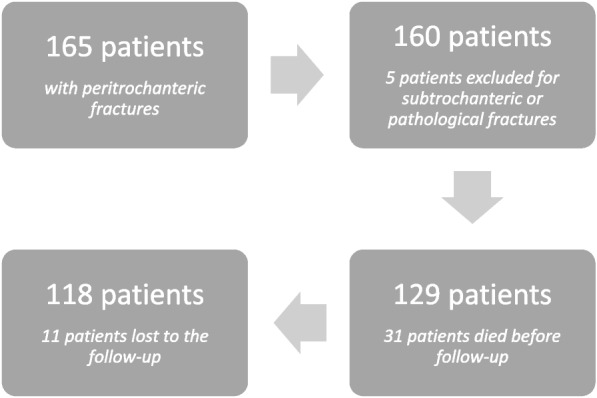
Flow chart diagram for the study’s cohort

A total of 165 patients were treated for peri-trochanteric fracture with intramedullary nailing during the study period in the same hospital. Of these patients, 47 were excluded from the study (31 died before the 3-months follow-up, 11 were lost to the follow-up and 5 for other reasons such as pathological fracture etc.). Figure 2 presents the inclusion process as a flow chart. After exclusions a total 118 patients were finally included in the study. The mean age was 82.7 years (range 50–97 years) and all of the patients sustained the fracture due to a low impact fall from standing height. The fractures were classified using the Jensen and the AO classification. Most of the patients had Jensen type 2 fracture (AO 31A1.3 & 31A2.1) (46/118) followed by the type 4 ones (AO 31A2.2) (28/118).

All patients were treated with a short proximal femoral intramedullary nail and randomly divided into two groups. In the group A, 54 patients were operated using the standard lag screw into the femoral head and in the group, 64 patient were operated using an additional anti-rotational lag screw. The two formed groups were comparable in terms of their age (mean age 82.3 for group A and 83.1 for group B, *p* > 0.05).

There were no statistically significant differences pre-operatively between the two groups regarding the ASA grade and the mental score (ASA grade 2.4 and 2.5 respectively and mental score 7.8 and 6.8 respectively with *p* > 0.05). Intra-operatively the two groups were not statistically significantly different in terms of operation time, transfusion rate and operative radiation dose and time (*p* > 0.05). The second group with the additional anti-rotational screw had slightly longer operation time (40 min against 30 min) but slightly less radiation time (0.3 versus 0.4 min). The mean transfusion rate was 1,7 units for group A and 1.8 units for group B. Similar were the results in terms of radiation dose with 3.2mAs for group A and 3.0mAs for group B. The pre-operative patient reported outcome measures (Nottingham Hip Fracture Score and Koval Score) were similar in the two groups with *p* > 0.05 (4 and 4.28 for the Nottingham Hip Fracture Score respectively and 3.4 and 3.5 for the Koval score respectively). Table 1 summarizes these results. There were significantly more complications observed in group A at the three months follow-up (*p* < 0.05). Group A had total 19 complications (3 cutouts, 6 with varus angulation, 8 ectopic ossification, one metalwork failure and one delayed union) and group B had only 5 complications (3 ectopic ossifications, one metalwork failure and one delayed union). Table 2 summarises the complication results. The z-effect was not observed in any cases. The radiological measurements are summarized in Table 3.

**Table 1:** Operative and follow-up data of the two study groups

	**Transfusion** *(units)*	**Radiation** *(Time/Dose)*	**Mental score**	**Operative time** *(mins)*	**ASA Grade**	**Nottingham Hip fracture score**	**Koval Score**
Group A	1,7	0,4 min 3,2mAs	7,8	30 min	2,4	4	3,4
Group B	1,8	0,3 min 3mAs	6,8	40 min	2,5	4,28	3,5

**Table 2:** Complications observed in the two groups

	**Cutout**	**Varus Angulation**	**Metalwork Breakage**	**Ectopic Ossification**	**Delayed Union**	**Total**
Group A	3	6	1	8	1	19
Group B	0	1	0	3	1	5

**Table 3:** Radiographic measurements in the two groups

	**Group A**	**Group B**	*p*
Ω	35mm	35,07mm	*n.s*
Ω1	13.89mm	14.05mm	*n.s*
ΤAD AP LS	11.69mm	10.54mm	*n.s*
TAD Ax Ls	10.675	11.05	*n.s*
Cervical Angle	133.134^o^	133.42^o^	*n.s*
Reduction Gap	3.584mm	3.17	*n.s*
X angle	1° positive	0.57° negative	*n.s*

**Fig. 3:**
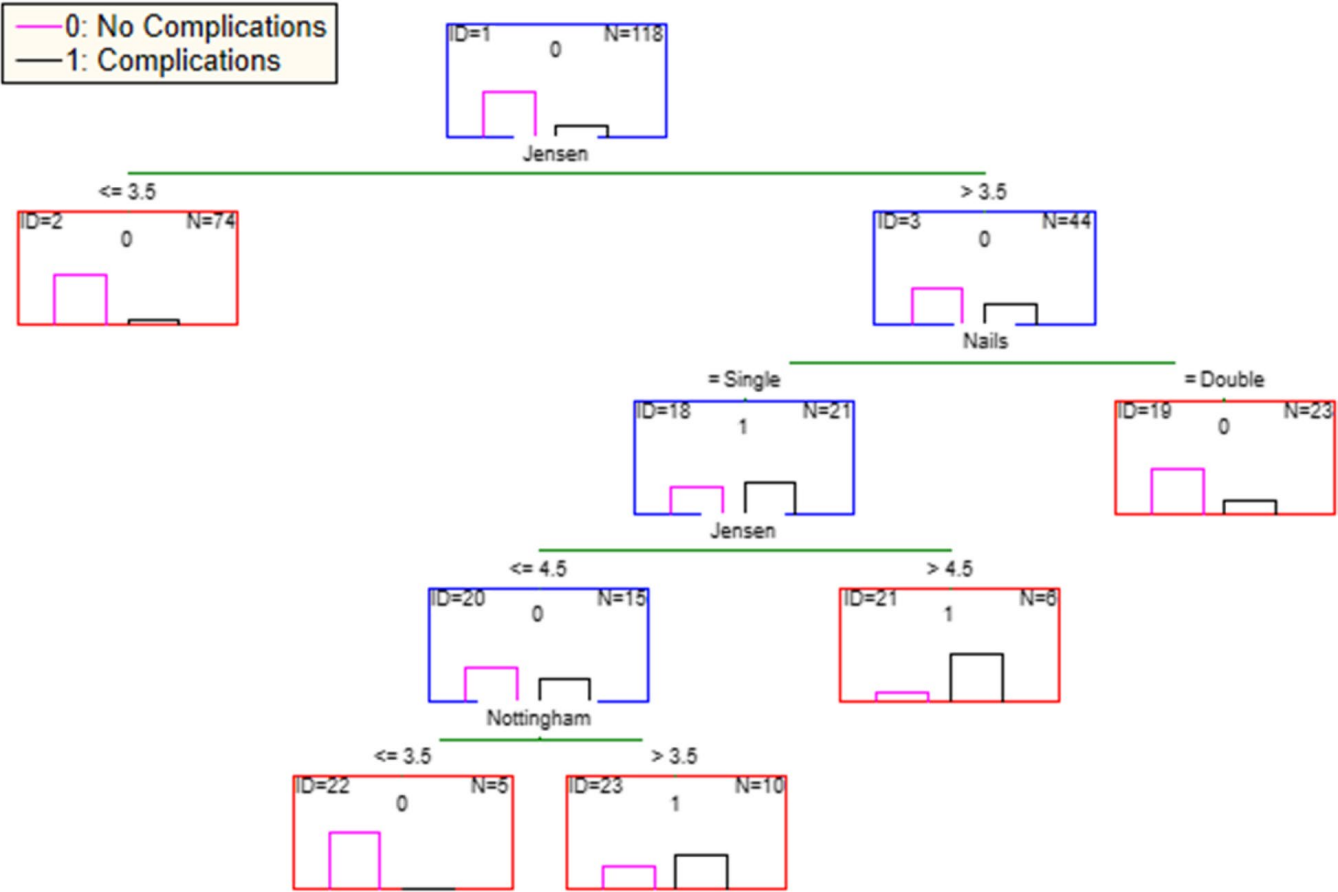
Classification Tree results

The CART analysis resulted in a tree with nine nodes four of which were terminal nodes (Fig. 3). The correct classification rate was 85.6%, exceeding the respective rate of the null model. Fracture severity appears to be a significant factor determining the possibility of post-surgery complications, with patients of fracture severity below three, according to the Jensen classification, having no complications, regardless the number of lag screws used. When the Jensen score, however, exceeds four (44 cases), the use of an additional anti-rotational lag screw appears to ensure largely the absence of post-surgery complications. This becomes even more obvious when the severity of the fracture is very high (Jensen score 5), where out of the six patients where a single lag screw was used, five experienced post-surgery complications.

The Chi-square analysis performed for the 44 cases with Jensen score exceeding the value of four (Table 4) confirmed the previous observation. The observed values of complications are higher than the predicted when one lag screw is used. The observed values of no complications are lower than the predicted when an anti-rotational lag screw is used. Subsequently there is a statistically significant tendency of unstable fractures to develop post-operative complications when a single lag screw is used.

**Table 4:** Chi-square results for the 44 cases with Jensen score above 4. In parenthesis the predicted values are shown. Pearson Chi-square = 4.454, *p* < 0.05

Nails	No Complications	Complications
Group A	10 (13.36)	11 (7.64)
Group B	18 (14.64)	5 (8.36)
Total	28	16

## Discussion

The present study demonstrates that a second anti-rotational screw can be proven crucial for unstable peri-trochanteric hip fractures, in terms of preventing serious complications, such as cutout, metalwork failure and varus angulation. The operation time was slightly longer though, but not significantly increased and all the radiological parameters for the nail and screw placement were not statistically significantly different. In terms of follow-up, the patient reported outcome scores of the two groups were similar, illuminating that the presence of an anti-rotational screw does not affect the clinical outcome. Concluding that the addition of this additional lag screw is not affecting the other parameters of the intramedullary nail, but indeed contributes to the significant reduction of the severe complications of the unstable peri-trochanteric fractures classified as type 4 and 5 with the Jensen classification (AO 31A2.2 and higher).

The finding of the present study are in line with previous similar literature. A randomised clinical trial by Vidyadhara and Rao [7], the presence of a second anti-rotational lag screw in the femoral neck did not affect the clinical outcomes, but less sliding of the screws was present in the two lag screws construct group. In that study only unstable intertrochanteric fractures were included, and the tip apex index (TAD) of the two lag screws construct group was significantly lower as well.

The above finding are backed up by some biomechanical studies as well. In a recent biomechanical study by Veerasakul et al. the double lag screw construct demonstrated greater stability when compared with a single lag screw construct [6]. In that study this difference in performance was present on fracture patterns without medial continuity (lesser trochanter absent), which is in line with the findings of the present study, as the two lag screw constructs were proven more reliable for the unstable fracture patterns (Jensen type 4&5). In another biomechanical study the two lag screw construct was proven to withstand higher loads when the lag screws were placed with higher TAD [19].

It is reported that peri-trochanteric fractures with significant lateral wall fragmentation can be treated with modern anatomic proximal femoral plates. These allow a reduced fluoroscopic time and intraoperative blood lose, without affecting the recovery and outcomes, when compared with the proximal intramedullary femoral nails [20]. The present study demonstrates that the use of a second anti-rotational lag screw through the nail for these unstable fractures can prevent most of the complications and the presence of the second anti-rotational screw in the upper part of the neck restores the continuity of this part, transfers the loads from the head to the central part of the femoral nail and guides the upper head and neck as it slides to anchor onto the central femoral nail. Also, the second lag screw, which is more centrally located, can immediately stabilize large pieces in the area of the lateral femoral wall as well.

The results of the present study are in line with other previous studies, which emphasize that the correct placement of the basic intertrochanteric heel in terms of position in the "safe zone" and the correct cephalocaudal direction are the most important factors to avoid complications [14]. In the present study the metalwork placement met these criteria, nevertheless, in some Group A cases (single lag screw) despite the correct placement of the metalwork, complications were observed. Similar fracture patterns treated with a second anti-rotational screw, remained stable, avoiding complications. Therefore, the correct placement of the metalwork alongside with the presence of the anti-rotational screw makes the system more reliable and more stable.

**Fig. 4:**
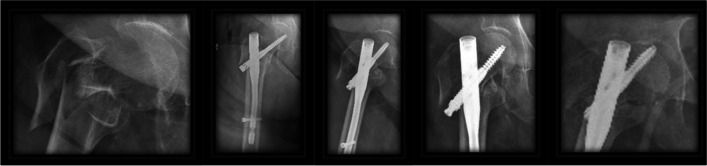
Sequential radiographs of a case from group A lead to cutout

**Fig. 5:**
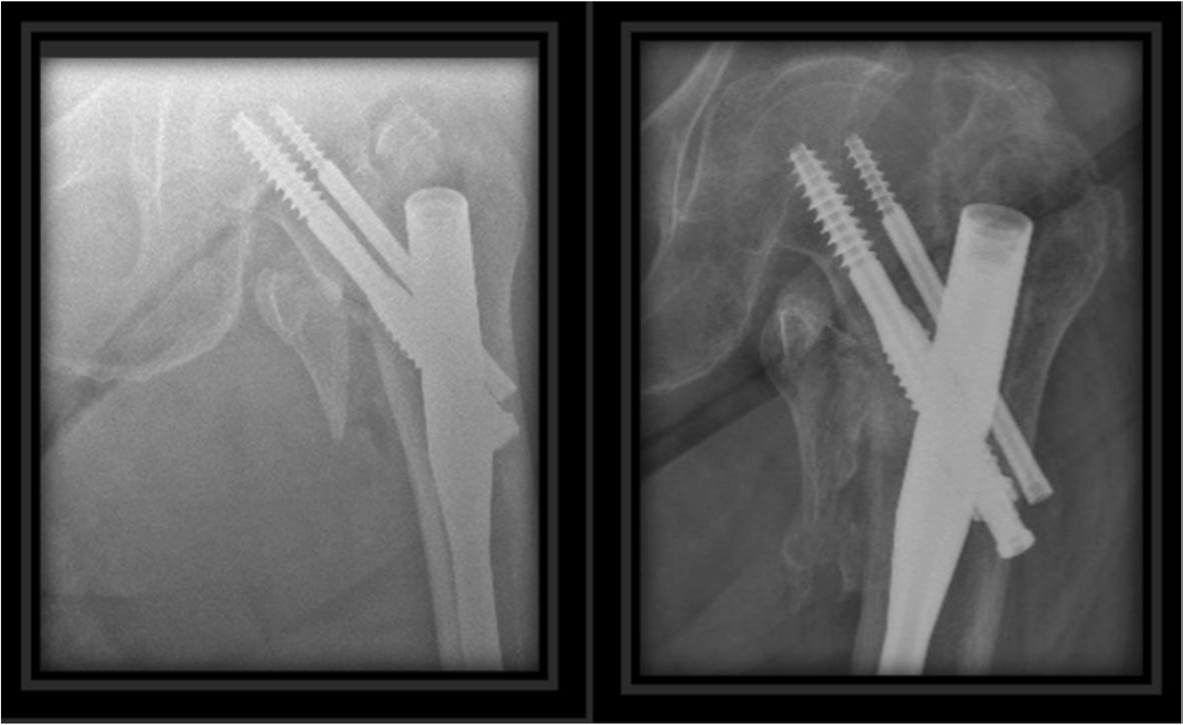
Sequential radiographs of a case with screw sliding from group B

The presence of the anti-rotational screw does not seem to prevent the posterior slip, but it does seem to make the slip more balanced. In the case of a single lag screw, while the lag screw slides, the head must also slide equally from all sides, if while the lag screw slides the head encounters resistance on one side. That side will stop sliding, so as long as the sliding continues from the opposite side of the head then the head will begin to move in relation to the lag screw with the possibility of leading to a cut-out. Figure 4 demonstrates the sequential progression of a case in group A, which led to a cut-out. In the cases where the second lag screw is present in the neck, the head may encounter resistance on one side and will provide an additional obstacle that does not allow the head to move in relation to the lag screws which should prevent the cut-out. Figure 5 demonstrate such case in group B.

The present study demonstrates several strengths such as the presence of only the second anti-rotational screw, as the only difference between the two study groups. The study groups were formed randomly but they were similar to many parameters. The limitations of the present study are the relative small number of cases included from a single centre, and the relative small follow up of the cases. Multicentre studies with larger cohorts will help to derive more generalized conclusions. Also the quality of reduction could have affected the results of the study, as it is of paramount importance for uneventful healing. All surgeons achieved good reduction before applying the intramedullary nail though, minimising this possibility.

## Conclusions

The use of proximal femoral intramedullary nail with a single lag screw for unstable peri-trochanteric fractures may lead to complications of varying levels of severity. Some could reach severe levels such as cut-out. The use of an additional anti-rotational lag screw for these fractures could prevent these type of complications and the treating orthopaedic surgeons should consider applying this configuration for the unstable fractures (Jensen type 4 and 5 or AO 31A2.2 and higher).

## Data Availability

The datasets used and/or analysed during the current study available from the corresponding author on reasonable request.
